# Supplementary material for: Establishment of a Quadruplex RT-qPCR for the Detection of Canine Coronavirus, Canine Respiratory Coronavirus, Canine Adenovirus Type 2, and Canine Norovirus
Source: Pathogens. 2024 Nov 29;13(12):1054. doi: 10.3390/pathogens13121054 (PMC11728440; doi:10.3390/pathogens13121054)
Supplement: Supplementary file 1 [file pathogens-13-01054-s001.zip › pathogens-3331081-supplementary.pdf]

Supplementary Materials

Table S1. The information on the reference strains of CCoV (7 strains of CCoV-I, 21 strains of CCoV-IIa, and 8 strains of CCoV-IIb)

Isolate	GenBank	Country	Collection Date	Submission Date	Genotype
12/20	OX335549.1	United Kingdom	/	13 Sep, 2022	I
62/22	OX335548.1	United Kingdom	/	13 Sep, 2022	I
59/22	OX335546.1	United Kingdom	/	13 Sep, 2022	I
10/22	OX335534.1	United Kingdom	/	13 Sep, 2022	I
46/22	OX335530.1	United Kingdom	/	13 Sep, 2022	I
38/22	OX335545.1	United Kingdom	/	13 Sep, 2022	I
13/22	OX335535.1	United Kingdom	/	13 Sep, 2022	I
450/07	GU146061.1	Italy	Oct, 2007	30 Oct, 2009	IIb
66/09AY390344	HQ450376.1	Greece	2009	28 Oct, 2010	IIb
CCoV/7/2020/AUS	MW383487.1	Australia	12 Mar, 2020	6 Dec, 2020	IIb
174/06	EU856362.1	Italy	Mar, 2006	27 Jun, 2008	IIb
341/05	EU856361.1	Italy	Dec, 2005	27 Jun, 2008	IIb
119/08	EU924791.1	Italy	Mar, 2008	25 Jun, 2008	IIb
Z19	MZ420153.1	Haiti	15 Mar, 2017	18 Jun, 2021	IIb
CoV/dog/HCM47/2015	LC190907.1	Vietnam	Jun, 2015	11 Oct, 2016	IIb
CB/05	KP981644.1	Italy	2005	16 Mar, 2015	IIa
NA/09	JF682842.1	Greece	2009	13 Mar, 2011	IIa
JS2103	OM055788.1	China	10 Mar, 2021	28 Dec, 2021	IIa
S378	KC175341.1	USA: Ithaca, NY	1978	15 Nov, 2012	IIa
1-71	JQ404409.1	USA	/	11 Jan, 2012	IIa
HC2	AY884048.1	China	/	11 Jan, 2005	IIa
SH32_2001	MF095847.1	Tanzania	2001	11 May, 2017	IIa
fc1	AB781790.1	Japan	/	7 Feb, 2013	IIa
04-0709	FJ009114.1	United Kingdom	/	11 Aug, 2008	IIa
B639_ZJ_2019	MT114538.1	China	19 Sep, 2019	25 Feb, 2020	IIa
CCoV/GD/2020/X9	MZ320954.1	China	Feb, 2020	31 May, 2021	IIa
GH8-2	OM950728.1	China	2020	8 Mar, 2022	IIa
UPPS2/04	DQ431019.1	Sweden	/	3 Mar, 2006	IIa
NS-28	MT136074.1	China	9 Mar, 2018	2 Mar, 2020	IIa
A76	JN856008.1	USA: Ithaca, NY	1976	16 Nov, 2012	IIa
2020/7	MT906865.1	United Kingdom	2020	20 Aug, 2020	IIa
742	KF308994.1	Brazil	2006	1 Jul, 2013	IIa
CD25_21032412	OQ623725.1	China	24 Mar, 2021	12 Mar, 2023	IIa
JS1706	MN078152.1	China	2017	17 Jun, 2019	IIa
CCoV/GY0608	MW718811.1	China	2020	5 Mar, 2021	IIa
859	KF308995.1	Brazil	2007	1 Jul, 2013	IIa

Table S2. The information on the reference strains of CRCoV (31 strains of CRCoV)

Isolate	GenBank	Country	Collection Date	Submission Date
PP014_THA_2013	OQ621712.1	Thailand	2013	14 Mar, 2023
PP106_THA_2014	OQ621711.1	Thailand	2014	14 Mar, 2023
PP049_THA_2014	OQ621710.1	Thailand	2014	14 Mar, 2023
PP010_THA_2013	OQ621709.1	Thailand	2013	14 Mar, 2023
PP008_THA_2013	OQ621708.1	Thailand	2013	14 Mar, 2023
PP007_THA_2013	OQ621707.1	Thailand	2013	14 Mar, 2023
BJ232	KX432213.1	China	2014	21 Jun, 2016
K37	JX860640.1	South Korea	2008	24 Sep, 2012
K9	GQ918141.1	South Korea	2008	16 Sep, 2009
K37	GQ918142.1	South Korea	2008	16 Sep, 2009
K39	GQ918143.1	South Korea	2008	16 Sep, 2009
4182	DQ682406.1	United Kingdom	2003	9 Jun, 2006
240/05	EU999954.1	Italy	2005	6 Aug, 2008
CRCoV/BJ-221	KR265207.1	China	2014	24 Apr, 2015
CRCoV/BJ-202	KR265206.1	China	2014	24 Apr, 2015
CRCoV/BJ-34	KR265205.1	China	2014	24 Apr, 2015
CRCoV-BJ202	KT852998.1	China	Jan, 2014	30 Sep, 2015
D153NS_THA_2021	OQ621727.1	Thailand	2021	14 Mar, 2023
D200NS_THA_2022	OQ621726.1	Thailand	2022	14 Mar, 2023
D197NS_THA_2022	OQ621725.1	Thailand	2022	14 Mar, 2023
D187NS_THA_2022	OQ621724.1	Thailand	2022	14 Mar, 2023
D206NS_THA_2022	OQ621723.1	Thailand	2022	14 Mar, 2023
D155NS_THA_2021	OQ621722.1	Thailand	2021	14 Mar, 2023
D154NS_THA_2021	OQ621721.1	Thailand	2021	14 Mar, 2023
D161NS_THA_2021	OQ621720.1	Thailand	2021	14 Mar, 2023
D177NS_THA_2022	OQ621719.1	Thailand	2022	14 Mar, 2023
D175NS_THA_2022	OQ621718.1	Thailand	2022	14 Mar, 2023
D140NS_THA_2021	OQ621717.1	Thailand	2021	14 Mar, 2023
D144NS_THA_2021	OQ621716.1	Thailand	2021	14 Mar, 2023
D057NS_THA_2021	OQ621715.1	Thailand	2021	14 Mar, 2023
PP158_THA_2015	OQ621714.1	Thailand	2015	14 Mar, 2023

Table S3. The information on the reference strains of CAV-2 (44 strains)

Isolate	GenBank	Country	Collection Date	Submission Date
CH-HN-1903	MN402899.1	China	9 Jul, 2019	3 Sep, 2019
CH-HN-1904	MN402900.1	China	3 Aug, 2019	3 Sep, 2019
CH-HB-1701	MN402901.1	China	11 Aug, 2017	3 Sep, 2019
CH-HB-1801	MN402902.1	China	12 Jun, 2018	3 Sep, 2019
CH-HB-1803	MN402904.1	China	12 Jun, 2018	3 Sep, 2019
CH-HB-1901	MN402905.1	China	3 May, 2019	3 Sep, 2019
CH-JS-1701	MN402906.1	China	2 Jun, 2017	3 Sep, 2019
CH-JS-1802	MN402908.1	China	5 Aug, 2018	3 Sep, 2019
CH-JS-1803	MN402909.1	China	10 Aug, 2018	3 Sep, 2019
CH-JS-1901	MN402910.1	China	11 Jun, 2019	3 Sep, 2019
CH-JS-1902	MN402911.1	China	12 Jul, 2019	3 Sep, 2019
CAdV2-53/2011	MT193142.1	Italy	2011	16 Mar, 2020
CAdV2-55/2011	MT193143.1	Italy	2011	16 Mar, 2020
CAdV2-88/2011	MT193144.1	Italy	2011	16 Mar, 2020
CAdV2-258/2006	MT193145.1	Italy	2006	16 Mar, 2020
CAdV2-618/1995	MT193146.1	Italy	1995	16 Mar, 2020
CAdV2-687/1999	MT193147.1	Italy	1999	16 Mar, 2020
CAdV2-800/2009	MT193148.1	Italy	2009	16 Mar, 2020
CAdV2-1149/2015	MT193149.1	Italy	2015	16 Mar, 2020
SH2015	MT737967.1	China	2015	8 Jul, 2020
CAV-WH2016	MW116177.1	China	2016	9 Oct, 2020
452/2017	MW829200.1	Italy	2017	26 Mar, 2021
NWT-W85	OK546121.1	Canada: Northwest Territories	/	15 Oct, 2021
G1	OP060355.1	China	2021	23 Jul, 2022
CAV-HN45	OP618115.1	China	23 Aug, 2020	19 Sep, 2022
Korea/2020/18Ra-65	OP645073.1	South Korea	2020	14 Oct, 2022
/	DQ839392.1	India	/	7 Jul, 2006
YCA-18	EF508034.1	China	/	20 Mar, 2007
CC0710QB	EU717145.1	China	Oct, 2007	13 May, 2008
cc0710QZ	EU794687.1	China	Oct, 2007	3 Jun, 2008
A2	LC557011.1	Japan	/	17 Jun, 2020
602-07-2008-spleen	MF344666.1	Norway	2008	12 Jun, 2017
603-06-1997/98-liver	MF344667.1	Norway	1997/1998	12 Jun, 2017
603-07-1997/98-kidney	MF344668.1	Norway	1997/1998	12 Jun, 2017
603-10-1999/00-spleen	MF344669.1	Norway	1999/2000	12 Jun, 2017
603-12-2001/02-spleen	MF344670.1	Norway	2001/2002	12 Jun, 2017
603-13-1999/00-lymphnode	MF344671.1	Norway	1999/2000	12 Jun, 2017
874-2014-Tongue	MH105809.1	Italy	2014	22 Mar, 2018
Fox/466/2017/ITA	MH399790.1	Italy	Jul, 2017	29 May, 2018
CH-HN-1801	MN402894.1	China	4 Sep, 2018	3 Sep, 2019
CH-HN-1802	MN402895.1	China	8 Jul, 2018	3 Sep, 2019
CH-HN-1901	MN402897.1	China	3 Jun, 2019	3 Sep, 2019
CH-HN-1902	MN402898.1	China	4 Jul, 2019	3 Sep, 2019
18Ra-54	OP644981.1	South Korea	2018	13 Oct, 2022

Table S4. The information on the reference strains of CNV (33 strains)

Isolate	GenBank	Country	Collection Date	Submission Date	Genotype
Dog/M9/18/CH	MN901144.1	China	26 Oct, 2018	6 Jan, 2020	GVI
Dog/M19/18/CH	MN901143.1	China	12 Sep, 2018	6 Jan, 2020	GVI
Dog/2-1-16/18/CH	MN901142.1	China	22 Aug, 2018	6 Jan, 2020	GVI
Dog/3-3-19/18/CH	MN901141.1	China	13 Jun, 2018	6 Jan, 2020	GVI
Dog/2-3-19/18/CH	MN901140.1	China	12 Apr, 2018	6 Jan, 2020	GVI
Dog/3-1-4/18/CH	MN901139.1	China	25 Feb, 2018	6 Jan, 2020	GVI
Dog/3-1-11/17/CH	MN901138.1	China	19 Jul, 2017	6 Jan, 2020	GVI
Dog/3-2-15/17/CH	MN901137.1	China	23 Jul, 2017	6 Jan, 2020	GVI
Dog/3-3-1/17/CH	MN901136.1	China	17 Dec, 2017	6 Jan, 2020	GVI
Dog/3-3-2/17/CH	MN901135.1	China	8 Dec, 2017	6 Jan, 2020	GVI
Dog/H3/19/CH	MN901134.1	China	20 Feb, 2019	6 Jan, 2020	GVI
Dog/3-3-3/17/CH	MN901133.1	China	17 Oct, 2017	6 Jan, 2020	GVI
Dog/3-3-4/17/CH	MN901132.1	China	12 Sep, 2017	6 Jan, 2020	GVI
Dog/3-3-5/17/CH	MN901131.1	China	28 Aug, 2017	6 Jan, 2020	GVI
Dog/3-3-6/17/CH	MN901130.1	China	13 Aug, 2017	6 Jan, 2020	GVI
Dog/3-3-19/17/CH	MN901129.1	China	9 Jul, 2017	6 Jan, 2020	GVI
Dog/W14/19/CH	MN901128.1	China	11 Jun, 2019	6 Jan, 2020	GVI
63.15/2015/ITA	KY486329.1	Italy	2005	18 Jan, 2017	GVI
dog/GVI.2/AN1640/USA/2017	MK067295.1	USA	2017	11 Oct, 2018	GVI
Dog/GVI.2/W14/19/CH	MN897757.1	China	11Jun, 2019	3 Jan, 2020	GVI
Dog/GVI.2/M19/18/CH	MN897758.1	China	12 Sep, 2018	3 Jan, 2020	GVI
Dog/GVI.2/M9/18/CH	MN897759.1	China	26 Oct, 2018	3 Jan, 2020	GVI
Dog/GVI.2/H3/19/CH	MN897760.1	China	20 Feb, 2019	3 Jan, 2020	GVI
Dog/GVI.2/M9/18/CH	MN908340.1	China	26 Oct, 2018	3 Jan, 2020	GVI
BUCT-K1	MW939576.1	China	9 Nov, 2020	16 Apr, 2021	GVI
BUCT-K4	MW945229.1	China	9 Nov, 2020	17 Apr, 2021	GVI
dog/FD210/2007/Ita	JF939046.1	United Kingdom	2007	11 May, 2011	GVI
Bari/91/07/ITA	FJ875027.1	Italy	2007	31 Mar, 2009	GVI
Dog/C19/19/CH	MN901151.1	China	21 Mar, 2019	6 Jan, 2020	GIV
dog/170/07/Ita	EU224456.1	Italy	/	17 Oct, 2007	GIV
Dog/GIV.2/C19/19/CH	MN912824.1	China	21 Mar, 2019	9 Jan, 2020	GIV
Dog/D42/19/CH	MW165826.1	China	5 Sep, 2019	26 Oct, 2020	GIV
dog/GIV.2/AN843/USA/2011	MK067289.1	USA	2011	11 Oct, 2018	GIV

Supplementary Materials

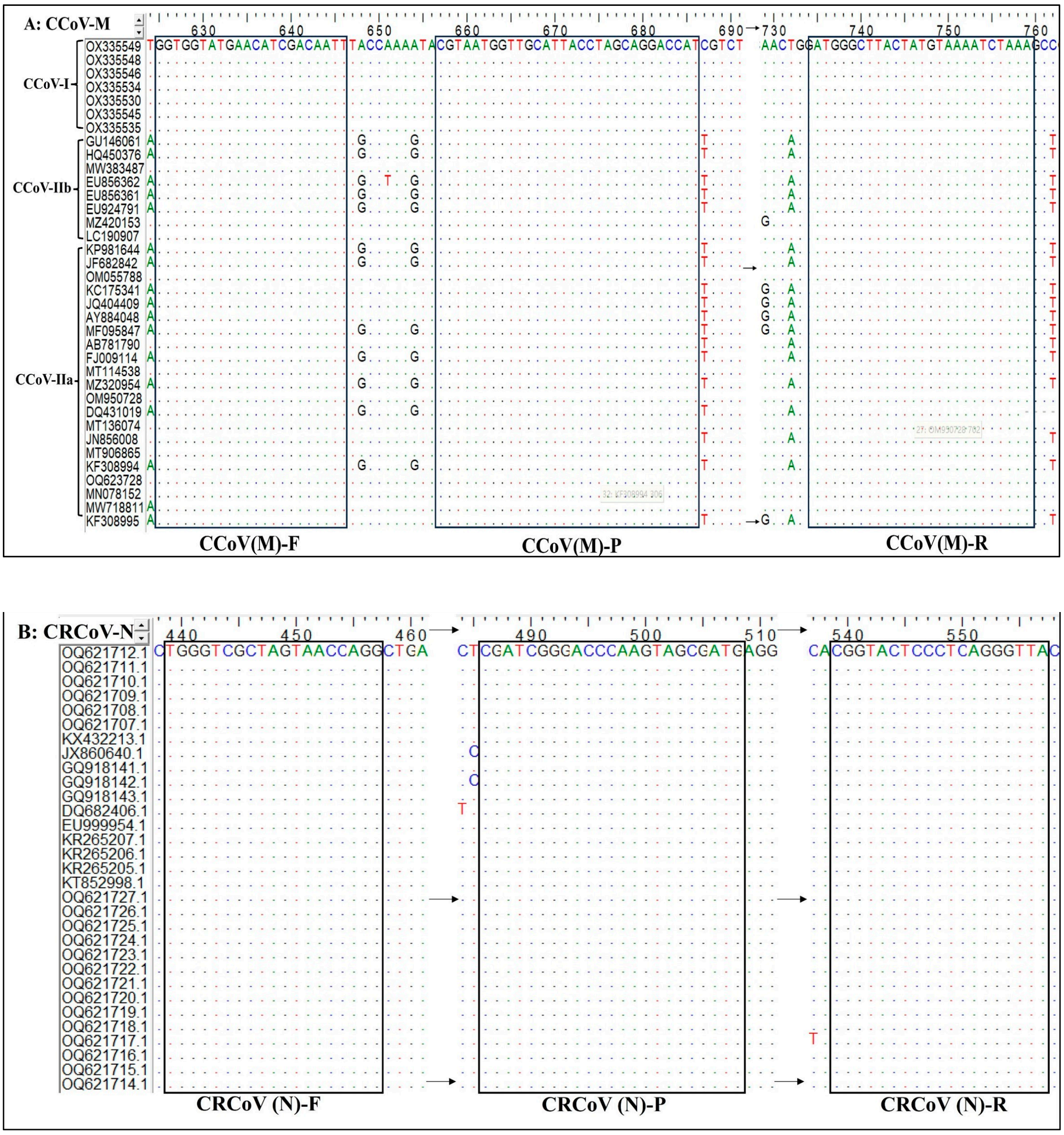

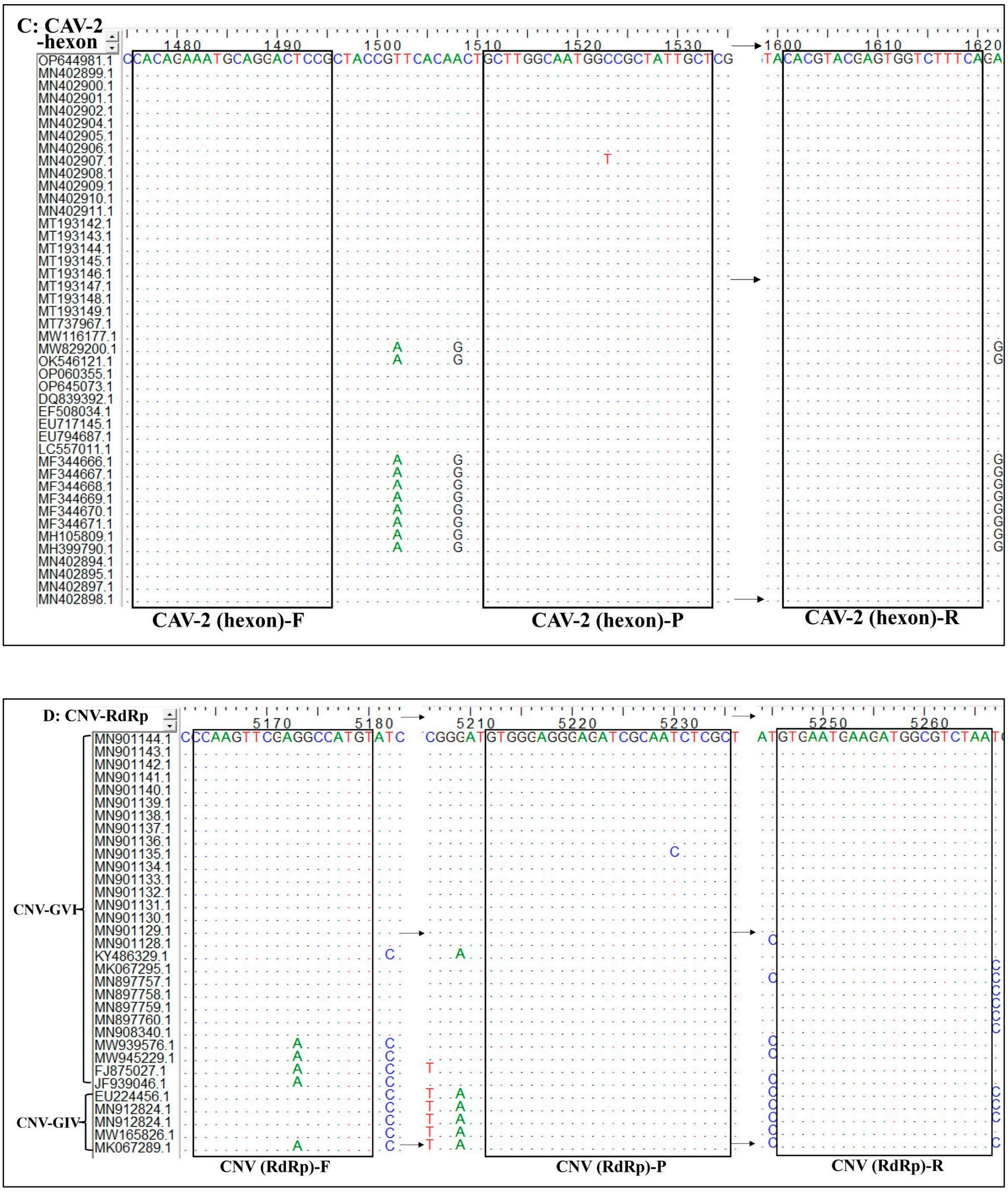

Figure S1. The locations of the specific primers and probes. The nucleotide sequence alignments indicate the partial CCoV M gene (A), CRCoV N gene (B), CAV-2 hexon gene (C), and CNV RdRp gene (D). F/R: forward/reverse primer; P: TaqMan probe.
